# Critical thresholds for intracranial pressure vary over time in non-craniectomised traumatic brain injury patients

**DOI:** 10.1007/s00701-018-3555-3

**Published:** 2018-05-07

**Authors:** Basil Nourallah, Frederick A. Zeiler, Leanne Calviello, Peter Smielewski, Marek Czosnyka, David K. Menon

**Affiliations:** 1 Division of Anaesthesia, Addenbrooke’s Hospital, University of Cambridge, Cambridge, UK; 2 Department of Surgery, Rady Faculty of Health Sciences, University of Manitoba, Winnipeg, Canada; 3 Clinician Investigator Program, Rady Faculty of Health Sciences, University of Manitoba, Winnipeg, Canada; 4 Section of Brain Physics, Division of Neurosurgery, Addenbrooke’s Hospital, University of Cambridge, Cambridge, UK; 5 Institute of Electronic Systems, Warsaw University of Technology, Warsaw, Poland

**Keywords:** Neuromonitoring, Neurocritical care, Traumatic brain injury, Threshold, Intracranial pressure, Cerebral perfusion pressure

## Abstract

**Background:**

Intracranial pressure (ICP)- and cerebral perfusion pressure (CPP)-guided therapy is central to neurocritical care for traumatic brain injury (TBI) patients. We sought to identify time-dependent critical thresholds for mortality and unfavourable outcome for ICP and CPP in non-craniectomised TBI patients.

**Methods:**

This is a retrospective cohort study of 355 patients with moderate-to-severe TBI who received ICP monitoring and were managed without decompressive craniectomy in a tertiary hospital neurocritical care unit. Patients were grouped in 2 × 2 tables according to survival/death or favourable/unfavourable outcomes at 6 months and serial thresholds of mean ICP and CPP, using increments of 0.1 and 0.5 mmHg respectively. Sequential chi-square analysis was performed, and the thresholds yielding the highest chi-square test statistic were taken as having the best discriminative value for outcome. This process was repeated over monitoring periods of 1, 3, 5 and 7 days and for each day of recording to establish time-dependent thresholds. The same analysis was performed for age and sex subgroups.

**Results:**

Global ICP thresholds were 21.3 and 20.5 mmHg for mortality and unfavourable outcome respectively (*p* < 0.001). After the first day of ICP monitoring, ICP thresholds fell to between 15 and 20 mmHg and remained significant (*p* < 0.05). Significant time-dependent CPP thresholds for mortality or unfavourable outcome were often not identified, and no identifiable trends were produced.

**Conclusion:**

Critical ICP thresholds in non-craniectomised TBI patients vary with time and fall below established ICP targets after the first day of monitoring.

**Electronic supplementary material:**

The online version of this article (10.1007/s00701-018-3555-3) contains supplementary material, which is available to authorized users.

## Introduction

Intracranial pressure (ICP) monitoring is a fundamental strategy in neurocritical care for traumatic brain injury (TBI) and is used routinely to guide medical and surgical intervention. Though well-supported by retrospective [1, 11, 12] and prospective studies [24], there is no level I evidence for outcome benefit from treatment guided by ICP monitoring. The only randomised controlled trial showed no improvement in outcome associated with maintaining monitored ICP below 20 mmHg compared to treatment guided by clinical and radiological assessment. Among many worthwhile caveats, the authors note that the lack of demonstrable efficacy may be attributed in part to the use of a generic, universal target ICP [9] and poor monitoring method (instant end-hour values).

Intracranial hypertension is commonly defined at a threshold of 20–25 mmHg in clinical practice, and unfavourable outcomes have been described at ICP thresholds ranging from 15 to 25 mmHg [5, 10, 16, 20]. Recent consensus guidelines from the Brain Trauma Foundation (BTF) specify a critical ICP threshold of 22 mmHg below which ICP should be maintained [8], based on evidence that ICP above this value best predicts mortality and severe morbidity [22].

However, it has been suggested that optimal ICP thresholds may vary with demographic characteristics such as age and sex [22]. It also stands to reason that patients treated medically may have different ICP thresholds than those treated with decompressive craniectomy (DC), as these groups experience distinct intracranial biomechanical parameters. Other physiological variables, for example, state of autoregulation, probably play additional roles [14]. Thus, applying a universal threshold to ICP management fails to account for the heterogeneity in patient-specific injury patterns and response to treatment interventions. Of particular importance, the 22-mHg threshold is derived from a mixed cohort of patients managed with and without DC. Surprisingly low time-dependent ICP thresholds for outcome have recently been described in DC patients [19], but time-dependent ICP threshold analysis has not been conducted in an exclusive cohort of patients managed without DC (non-DC).

Similarly, BTF guidelines recommend targeting cerebral perfusion pressure (CPP) between 60 and 70 mmHg based on available evidence [2, 8, 22], but note the growing consensus that individualised cerebral perfusion pressure thresholds aimed at achieving optimal cerebrovascular reactivity may be associated with superior outcomes [3, 23]. The same may be true of ‘critical threshold’ of ICP [14]—although this point is much worse documented.

Thus, there is a need to identify more precise ICP and CPP thresholds that account for individual variation in age, sex, disease natural history and treatment intervention. The primary aim of this study is to evaluate, in a non-DC TBI patient population, ICP and CPP thresholds associated with 6-month morbidity and mortality. Further, we wish to evaluate whether these thresholds vary based on the recording period utilised, or based on the day of recording. Finally, secondary aims were to preliminarily assess if these critical thresholds vary based on age and sex.

## Methods

### Patient population

This is an observational cohort study with retrospective analysis of 355 patients within a TBI database. A portion of this population has been described in the previous ICP threshold work done by Sorrentino et al. [22]. These patients were those with a minimum of 6 h of archived high-frequency physiologic recordings. All patients were admitted to the Neurosciences and Trauma Critical Care Unit (NCCU) at Cambridge University Hospitals NHS Foundation Trust (CUH) between March 2005 and December 2016. Patients suffered either moderate-to-severe TBI or mild TBI and subsequently deteriorated to a point where they required ICP monitoring and sedation and mechanical ventilation as part of ICP management. As such, the timing of ICP monitoring, both in duration and time to initiation after injury, was variable. Treatment received included standard ICP-directed therapy, with an ICP goal of less than 20 mmHg and CPP goal of greater than 60 mmHg.

Age, sex, Glasgow Coma Scale (GCS) at admission, injury severity score (ISS) and Glasgow Outcome Scale (GOS) at 6 months were prospectively collected and recorded in the database. Two binary outcome classifications were recorded for each patient at 6 months, namely, alive vs dead and favourable vs unfavourable outcome. Unfavourable outcome was defined as GOS < 4.

All data were fully anonymised, and no attempt was made to re-access clinical records for additional information. As such, formal patient or proxy consent was not required. Within our institution, patient data may be collected with a waiver of formal consent, as long as it remains fully anonymised, with no method of tracing this back to an individual patient. Patient physiologic, demographic and outcome data were collected by the clinicians involved with patient care and subsequently recorded in an anonymous format. This anonymous data is then provided for future research purposes. Such data curation remains within compliance for research integrity as outlined in the Governance Arrangements for Research Ethics Committees (GAfREC), September 2011 guidelines, section 6.0 [17, 25].

The exact number of overlapping patients between the current work and Sorrentino et al. is unknown. As our local prospective signal database is a fluid entity, with patient data added at various points during their hospital stay, we are unable to determine which patients exactly overlap between these two works. In addition, we do not have access to the original anonymous patient identifier codes from the Sorrentino work, further impeding our ability to give exact numbers of overlap.

### Signal acquisition and processing

Intracranial pressure was acquired via an intraparenchymal strain gauge probe (Codman ICP MicroSensor; Codman & Shurtleff Inc., Raynham, MA). Arterial blood pressure was obtained through either radial or femoral lines connected to pressure transducers (Baxter Healthcare Corp. CardioVascular Group, Irvine, CA). All signals from the above devices were sampled at minimum 50 Hz and recorded using digital data transfer or digitised via an A/D converter (DT9801 or DT9803; Data Translation, Marlboro, MA), where appropriate, using the ICM+ software (Cambridge Enterprise Ltd., Cambridge, UK, http://icmplus.neurosurg.cam.ac.uk). Signal artefacts were removed manually prior to further processing or analysis. CPP was determined as the difference between mean arterial pressure and ICP.

Minute-by-minute data processed originally by ICM+ were exported into comma separated values format (CSV). Using R statistical software (R Core Team (2016). R: A language and environment for statistical computing. R Foundation for Statistical Computing, Vienna, Austria. URL https://www.R-project.org/), various data sheets were created including grand mean over entire recording period, first 24 h, first 72 h, first 120 h and first 168 h. Furthermore, data was also averaged for each day of recording in order to assess time dependence of the critical thresholds.

### Statistics

All statistics were completed using R statistical software. Descriptive statistics were applied to summarise demographic data. Normality was tested using the Shapiro-Wilk test for demographic variables and measured indices, all of which were determined to be non-parametric. Non-parametric tests of independence were used to compare demographic and clinical characteristics in patients of each binary outcome classification. Critical thresholds for outcome were derived using sequential chi-square analysis. This method has been previously applied in other publications assessing thresholds for ICP, CPP and continuous autoregulation indices in TBI [21, 22, 27], and its use allowed direct comparison of time-, duration- and treatment-dependent results with existing thresholds. Sequential 2 × 2 binary outcome contingency tables were constructed, grouping patients by (1) survival or dichotomised outcome (GOS ≥ 4 vs GOS < 4) and (2) average ICP and CPP greater or less than sequential thresholds in 0.1 and 0.5 mmHg increments respectively. The ICP or CPP threshold returning the highest chi-square test statistic value was assumed to have the best discriminative value.

The same analysis was repeated for each subgroup of sex (male vs female) and age (≤ 55 vs < 55). Age categories were set to mirror those used in the initial landmark threshold analysis [22]. Whole cohort and subgroup analysis was repeated for ICP and CPP values averaged over the first 24 h and 3, 5, 7 and 10 days of ICP monitoring to create duration-of-monitoring-dependent thresholds. Subsequently, the same analysis was repeated again for ICP and CPP means in 24 h slices to determine critical thresholds for each day of the first 7 days of ICP monitoring. Statistical significance was set at *p* < 0.05 for all results. *p* values were corrected for multiple testing using the Benjamini and Hochberg FDR method, and the results that remain statistically significant were highlighted. Graphical production was completed using the ggplot2 package in R.

Finally, univariable logistic regression analysis was performed for the statistically significant ICP thresholds, assessing the area under the receiver operating curve (AUC) and 95% confidence intervals (CI) associated with both dichotomised outcomes. This was conducted across all time periods analysed. This was not conducted for CPP, given poor threshold discrimination on chi-square testing.

## Results

### Cohort characteristics

A total of 355 patients (271 male, 84 female) were included in the study, with a mean age of 40.6 (SD 17.2) and a median admission GCS of 7 (IQR 3–9). The mean duration of ICP monitoring was 6.81 days (SD 5.99). Six months after admission, 172 patients had favourable outcomes as assessed by the Glasgow Outcome Score (low or moderate disability), and 183 had unfavourable outcomes (severe disability, persistent vegetative state or death) of whom 63 had died. Mean age and mean ICP were significantly higher among patients who died or had an unfavourable outcome. Mean ICP monitoring period was also higher in patients with unfavourable outcome. Median admission GCS was lower in patients who died or unfavourable outcome, while sex distribution, median ISS and mean CPP were not significantly different (Table 1). Mean ICP and CPP by patient outcome (alive/dead and favourable/unfavourable) for each day and duration of monitoring are given in Appendix A.

**Table 1:** Cohort characteristics

	Whole cohort	Favourable outcome (GOS ≥ 4)	Unfavourable outcome (GOS < 4)	*p* value	Survived	Died	*p* value
Number of patients	355	172	183		292	63	
Sex				0.76			0.27
Male Female	271 84	133 39	138 45		219 73	52 11	
Mean age (SD)	40.6 (17.2)	38.2 (17.0)	43.0 (17.0)	0.0091	39.4 (16.6)	46.6 (18.5)	0.0026
Mean ICP (mmHg) (SD)	14.1 (7.7)	12.6 (4.6)	15.4 (19.5)	0.0039	12.9 (5.8)	19.4 (12.0)	< 0.001
Mean CPP (mmHg) (SD)	77.5 (8.5)	77.6 (6.7)	77.6 (9.9)	0.31	77.9 (7.5)	76.2 (11.9)	0.39
Mean ICP monitoring period (days) (SD)	6.81 (5.99)	6.39 (5.50)	7.21 (6.38)	0.0030	6.95 (6.21)	6.21 (4.78)	0.31
Median admission GCS (IQR)	7 (3.25–9)	8 (5–11)	6 (3–8)	< 0.001	7 (4–10)	4 (3–8)	0.0026
Median ISS	33 (25–41)	31.5 (25–38)	33 (25–41)	0.20	33	29.5	0.76

*CPP* cerebral perfusion pressure, *GCS* Glasgow Coma Scale, *GOS* Glasgow Outcome Score, *ICP* intracranial pressure, *IQR* interquartile range, *ISS* injury severity score, *SD* standard deviation

### Overall thresholds

**Fig. 1:**
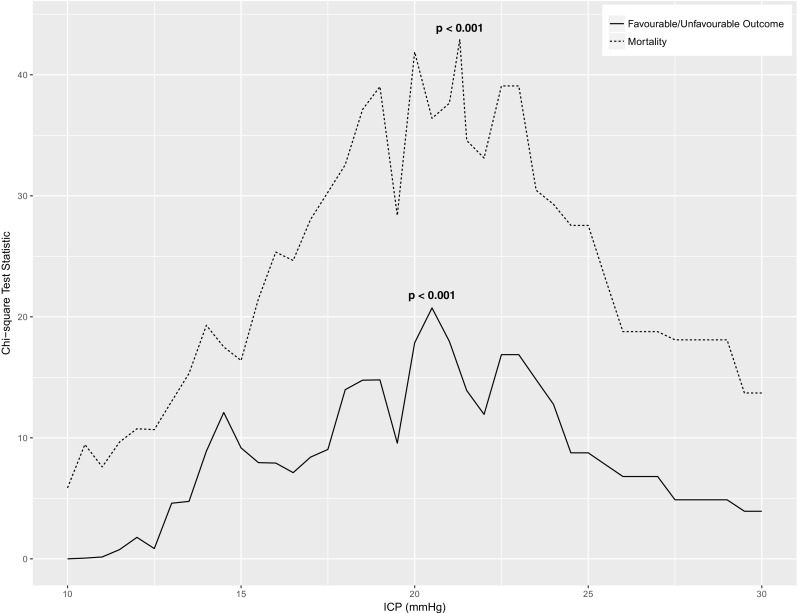
Sequential chi-square analysis for intracranial pressure threshold. The values returning the highest chi-square test statistic, and therefore having the highest discriminatory value for outcome, were taken as thresholds and are denoted by *p* values. Bold *p* values remained significant on correction for multiple comparisons. ICP denotes intracranial pressure

In the whole cohort across the full duration of ICP monitoring, the overall ICP threshold for mortality was 21.3 mmHg (*χ*^2^ = 42.90, *p* < 0.001). The threshold for the unfavourable outcome was 20.5 mmHg (*χ*^2^ = 20.73, *p* < 0.001) (Fig. 1).

The ICP thresholds for mortality in patients aged > 55 (*n* = 78) and ≤ 55 (*n* = 277) were 14.7 mmHg (*χ*^2^ = 8.3; *p* = 0.0039) and 20.7 mmHg (*χ*^2^ = 39.34; *p* < 0.001) respectively, while the thresholds for unfavourable outcome were 19.9 mmHg (*χ*^2^ = 4.97; *p* = 0.026) and 20.6 mmHg (*χ*^2^ = 17.35; *p* < 0.001) respectively. Male and female patients were also evaluated, but given the small number of female relative to male patients, the strength of conclusions is limited. Sex-specific results are presented in Appendix B. Univariable logistic regression analysis for the statistically significant thresholds can be found in Appendix C, providing AUC and 95% CI for the dichotomised outcomes across all statistically significant ICP thresholds identified.

### Duration-dependent thresholds

**Fig. 2:**
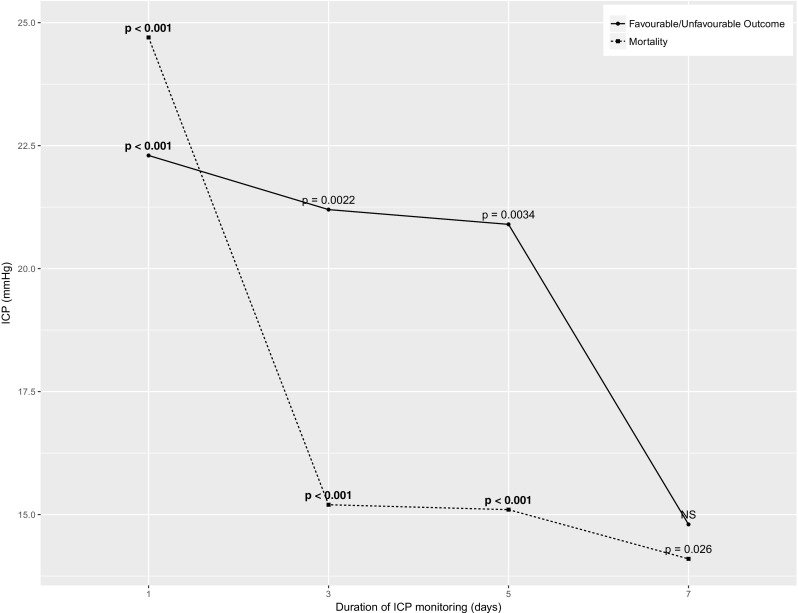
Whole cohort thresholds by duration of monitoring ICP. Bold *p* values remained significant on correction for multiple comparisons. Number of patients with available ICP data over each monitoring period were as follows: 24 h, 340; 3 days, 277; 5 days, 217; and 7 days, 163. ICP denotes intracranial pressure; NS not significant

Sequential chi-square analysis was repeated across all subgroups during the first 24 h and 3, 5 and 7 days of ICP monitoring. The whole cohort results are presented in Fig. 2. Over the first 24 h, the threshold for mortality was higher than the overall threshold (24.7 mmHg; *χ*^2^ = 31.30; *p* < 0.001) then dropped below 20 mmHg over longer periods of monitoring. The threshold for unfavourable outcome remained comparatively stable across all recorded lengths of monitoring, though significant unfavourable outcome thresholds could not be identified at 7.

The duration-dependent ICP thresholds for males and patients of age ≤ 55 closely followed the pattern of the overall cohort. The 24-h threshold for the unfavourable outcome was lower in female patients. Female mortality thresholds mirrored overall thresholds over 24 h and 3 days, but significant thresholds could not be identified beyond this (Appendix B). In older patients, lower thresholds were identified for both mortality and unfavourable outcome in the first 24 h, but further duration-dependent thresholds are not significant (Appendix D). It must be acknowledged that the number of patients included dropped over longer durations of recording.

### Time-dependent thresholds

**Fig. 3:**
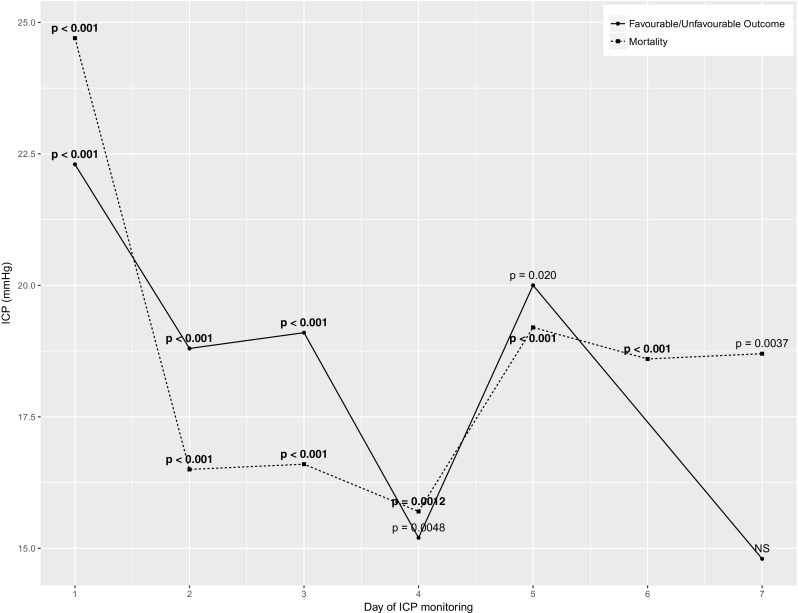
Whole cohort ICP thresholds by day of monitoring. Not significant ICP outcome point at 6 days removed. Bold *p* values remained significant on correction for multiple comparisons. Number of patients with available ICP data for each day of monitoring were as follows: day 1,340; day 2,296; day 3,270; day 4,234; day 5,199; day 6,176; and day 7,144. ICP denotes intracranial pressure; NS not significant

Sequential chi-square analysis was repeated for each 24-h period for the first 7 days of ICP monitoring. In the whole cohort, ICP threshold for mortality dropped from 24.7 mmHg (*χ*^2^ = 31.30; *p* < 0.001) in the first day to 16.5 mmHg (*χ*^2^ = 29.29; *p* < 0.001) in the second day and remained below 20 mmHg for each 24 h period assessed. The threshold for unfavourable outcome dropped from 20.3 mmHg (*χ*^2^ = 36.41; *p* < 0.001) on the first day to remain at or below 20 mmHg for each day where a significant threshold was identified (Fig. 3).

Time-dependent thresholds in the male and young subgroups were similar to overall cohort. Where significant thresholds were identified for the female subgroup, they were also similar with the exception of a lower day 1 outcome threshold (Appendix B). Patients aged > 55 had lower ICP thresholds for both mortality and unfavourable outcome for the first 2 days of monitoring, but significant thresholds were not identified for later days (Appendix D).

## Discussion

### ICP

This study presents the first systematic analysis of time-dependent ICP thresholds for mortality and unfavourable outcome in TBI patients managed without DC. The overall thresholds, based on the entire recording period, that best discriminate mortality and unfavourable outcome were 21.3 and 20.5 mmHg respectively. These thresholds were the most statistically significant ICP thresholds associated with global patient outcome, more so than a threshold of 20 (i.e. a commonly applied treatment threshold in clinical practice). They indicate that patients with mean ICP values above these particular thresholds had the strongest association with worse dichotomised global outcomes at 6 months. These are slightly below the universal threshold of 22 mmHg recommended in the current consensus guidelines [8], though they remain in the 20–25 mmHg range usually applied in clinical practice. However, time-specific analysis revealed variable ICP thresholds over the first week of monitoring. After the first 24 h of monitoring, the daily thresholds fell and remained between 15 and 20 mmHg. This was apparent both when the data was grouped by duration of monitoring and analysed on a day-by-day basis.

Sex-specific analysis revealed female patients had a higher overall ICP threshold for mortality, but the duration- and time-dependent ICP thresholds followed a similar pattern to those of male patients, with only occasional variations. Given the low number of female patients in the cohort, it is not possible to comment definitively on significant sex differences in ICP threshold based on this data alone. It is interesting to note lower ICP thresholds for mortality and unfavourable outcome in older patients, both overall and in the first 2 days of ICP monitoring. Though carrying a similar caveat due to the low proportion of older patients, this is in keeping with previous evidence that older patients may exhibit higher vulnerability to ICP insult [22], particularly in the early stages of ICP management. It is also notable that chi-square test statistic values are generally substantially higher in mortality thresholds for both ICP and CPP, suggesting a stronger association between these metrics and mortality as compared with the unfavourable outcome. This is concordant with previous results [22].

Our results establish ICP thresholds for non-DC patients that are slightly distinct from the threshold previously described for a mixed cohort [22]. Moreover, it is unsurprising to find these thresholds differ to those recently described for DC patients [19]. These results, taken together, reinforce the need to develop individualised ICP thresholds, as treatment modalities and patient characteristics introduce tremendous heterogeneity.

The ultimate goal is to derive ICP thresholds that derive rationally from patient-specific pathophysiology. There is evidence that the tolerability of ICP insults vary between patients depending on autoregulatory capacity [13] and intracranial pressure-volume dynamics [7]. Lazardis et al. [14] used a pressure reactivity index (PRx—correlation between ICP and MAP) cut-off of > 0.2 to define patient-specific ICP thresholds at which cerebrovascular pressure reactivity was deranged. These variable thresholds yielded superior outcome prediction than fixed thresholds of 20 or 25 mmHg. In the context of our findings, which show a time-dependent ICP threshold that usually remains below 20 mmHg, pulse amplitude index (PAx—correlation between pulse amplitude of ICP and MAP) may be superior for the latter strategy, as it is proven to be a superior outcome predictor than PRx at ICP < 20 mmHg [4].

### CPP

We explored CPP thresholds using the same methodology. The results showed no identifiable trends, and significant time-dependent thresholds for mortality or unfavourable outcome were often not identified, even in the whole cohort and in well-populated subgroups (males and young patients). As no significant conclusions could be drawn from these results, they have not been included in the manuscript.

This lack of ability to determine global clinically significant CPP thresholds likely stems from the issue of targeting a global CPP target for everyone. CPP thresholds in TBI have remained relatively elusive for some time now, with conflicting literature on appropriate CPP ranges to target, leading to changes in recommendations between various renditions of the BTF guidelines [6, 8]. Though the lack of significant results within this study does not provide definitive evidence, we believe they do provide further support of ‘personalised’ CPP targets in TBI. Such personalised CPP targets include those suggested by the CPP optimum (CPPopt) concept, with numerous papers to date suggesting a stronger link between CPPopt and patient outcome [18]. The definitive link between these personalised CPP targets in TBI has yet to be determined, with prospective randomised trials underway [COGITATE, ClinicalTrials.gov Identifier: NCT02982122].

We do not believe that the lack of results for CPP suggests that ICP is more important and should be preferentially targeted in TBI therapy. It is likely the way we currently view CPP is the issue, arguing for a change in the way we analyse and target it.

### Limitations

First, this study is limited by its retrospective design, making it impossible to control for the influence of treatment targets on the derived thresholds. Additionally, as high ICP may reflect the global severity of a patient’s condition, it is impossible to determine the direct effect of ICP thresholds on the outcome without prospective validation. Furthermore, as these data were collected over 11 years, changing management patterns over time may confound the results. Given the potential for treatment heterogeneity across our patient population, this needs further acknowledgement as a limitation. The information within our database was retrospectively accessed for the purpose of this study, without the ability to re-access charts for missing information. Thus, comments on treatment intensity (i.e. doses of hypertonic agents, use of sedation, cerebrospinal fluid drainage, use of barbiturates) and its potential relation to the thresholds seen cannot be made based on this data. This is an important limitation of our study, as not only can various treatments directly impact the ICP and CPP values recorded, but also those patients with medically refractory ICP may have had a reduction on therapy secondary to futility. Such a reduction in therapy could lead to persistent ICP elevations skewing the thresholds found within our study. However, with that said, we believe that the number of patients that would fall into this category would be quite small within the population studied, as those with persistent refractory ICP issues typically undergo a secondary DC at our institution. All DC patients were excluded from this study to avoid such confounding. Thus, despite the absence of information on treatment intensity, we are confident that the results presented are not significantly skewed due to outliers.

Furthermore, despite the exclusion of DC patients from our study, there were subjects in our study population that had medically refractory ICP (i.e. levels persistently above 20 mmHg during a large portion of their ICP stay). It is unknown why these patients did not undergo DC, as this was not recorded within our database that was retrospectively accessed for the purpose of this study. It is possible that these patients represent those that were ‘too sick’ for surgical salvage therapy. This further adds to the heterogeneity of our TBI population for this study, thus re-emphasising that the results found for ICP and CPP thresholds are preliminary, with further validation in a multicentre cohort required.

Second, although 355 patients were included in the study, the characteristic demographic pattern of TBI meant that the statistical power of less well-populated subgroup analysis was weakened by small sample size, notably in females and patients aged > 55. This may explain the relative absence of statistically significant thresholds in these subgroups especially. Thus, we were unable to definitively comment on whether there is a significant difference in ICP thresholds between age and sex groups. There exists the potential that age-related cerebral atrophy can confound the ICP thresholds seen. With advanced age, cerebral atrophy increases. Thus, to reach the same ICP values as younger patients, one could argue that more severe intracranial injury is required in these elderly patients, with larger mass lesions/oedema volume required. Thus, when assessing ICP thresholds associated with outcome, this potential injury severity discrepancy between the young and elderly, with similar ICP values, may impact the results. Based on the data available in our database, we were unable to assess this. We are planning a follow-up study with the ICU cohort from CENTER-TBI [15], using upwards of 2000 patients, with detailed imaging assessments, to probe into age-specific ICP thresholds and the impact of intracranial injury patterns.

Third, the time-dependent analysis is limited by values of ICP averaged over 24 h. Therefore, it was not possible in this work to identify time-dependent critical thresholds over a shorter timescale. Moreover, the average ICP values do not directly relate to time spent above putative thresholds, or ICP ‘dose’ [13, 26]. The drop in ICP-based critical thresholds after the first 24 h of recording may be secondary to either treatment effect, or reflect the ‘dose’ response to ICP, where absolute thresholds may be less useful than the amount of time spent above such values [13, 26]. While this generally did not have an effect on the derived ICP thresholds, the accuracy of the sequential chi-square method of threshold analysis is limited at extreme ICP values by small cell sizes.

Fourth, a further limitation of this retrospective database study is lack of clarity on cause of mortality. The information available within our dataset includes only a rough global assessment of patient outcome at 6-months (i.e. GOS), without elaboration on cause of mortality. Thus, we are unable to separate those who died secondary to neurological causes from other in-hospital causes within this study. This would have provided potentially interesting insight into differences in ICP and CPP thresholds based on mortality causes. Given this limitation within our study, this will be assessed in the upcoming analysis of the multicentre high-resolution ICU cohort in the CENTER-TBI [15], with the hope that further light may be shed on this topic.

Fifth, DC patients were specifically excluded to avoid the potential for confounding effects on the ICP thresholding. Much additional work is required in this population to assessed ICP and CPP critical thresholds associated with global outcome. In the presence of DC, the value returned from ICP monitoring is exceedingly difficult to interpret, given an open cranial vault and influence from atmospheric pressure on the readings. Thus, despite the difference in ICP threshold discovered between our work and the original Sorrentino study, we refrain from making generalised comments on ICP thresholds from DC patient populations at this time, until further objective analysis of this population is conducted. This work is currently under way.

Finally, it is important to emphasise that this work is preliminary. Thus, although we have identified differing trends within the non-DC population, and a change in threshold over time, further confirmatory work is required prior to any change in guideline-based threshold targets. We must re-emphasise that the results of this analysis should not change current ICP treatment thresholds recommended in current guidelines. In addition, having separate thresholds for mortality and favourable/unfavourable outcome is not entirely clinically useful, as it pertains to threshold targeting for ICP-directed therapies. These different thresholds only provide information regarding potentially predicting functional outcome. We plan to undertake further prospective analysis using the ICU cohort data-set from CENTER-TBI, to better define ICP thresholds over time and perform more detailed subgroup analysis.

## Conclusion

A global ICP threshold for non-DC TBI patients is identified as 21.3 mmHg for mortality and 20.5 mmHg for unfavourable outcome, similar to what has been described in the BTF guidelines. However, after the first day of ICP monitoring, time-dependent ICP thresholds fell to between 15 and 20 mmHg, lower than the targets recommended in current guidelines and used in clinical practice. These thresholds are lower still in older patients. These results underscore the importance of developing individualised ICP thresholds based on demographics, treatment modalities, patient-specific pathophysiology and, possibly, other physiological markers.

## Electronic supplementary material

ESM 1 (DOCX 97 kb)

ESM 2 (DOCX 222 kb)

ESM 3 (DOCX 135 kb)

ESM 4 (DOCX 210 kb)

ESM 5 (DOCX 120 kb)
