# Supplementary material for: Critical thresholds for intracranial pressure vary over time in non-craniectomised traumatic brain injury patients
Source: Acta Neurochir (Wien). 2018 May 7;160(7):1315–24. doi: 10.1007/s00701-018-3555-3 (PMC5996002; doi:10.1007/s00701-018-3555-3)
Supplement: Supplementary file 1 — (DOCX 97 kb) [file 701_2018_3555_MOESM1_ESM.docx]

|  | Mean ICP (sd) (mmHg) | | | | |  |
| --- | --- | --- | --- | --- | --- | --- |
|  | Alive | Dead | p | Favourable outcome | Unfavourable outcome | p |
| 1 day | 14.48 (6.29) | 20.91 (14.39) | < 0.001 | 13.93 (5.17) | 17.18 (5.17) | 0.003 |
| 3 days | 14.19 (5.96) | 18.68 (11.72) | < 0.001 | 13.69 (4.54) | 16.08 (9.15) | 0.022 |
| 5 days | 14.35 (4.78) | 18.61 (10.25) | < 0.001 | 13.89 (4.67) | 16.07 (7.19) | 0.015 |
| 7 days | 15.13 (4.52) | 16.78 (4.46) | 0.066 | 14.91 (4.23) | 15.80 (4.75) | 0.30 |

Appendix A

Table 1 By duration of monitoring: mean ICP by outcome at 6 months. ICP denotes intracranial pressure; sd standard deviation.

|  | Mean ICP (sd) (mmHg) | | | | | |
| --- | --- | --- | --- | --- | --- | --- |
|  | Alive | Dead | p | Favourable outcome | Unfavourable outcome | p |
| Day 1 | 14.48 (6.29) | 20.91 (14.39) | < 0.001 | 13.93 (5.17) | 17.18 (5.17) | 0.003 |
| Day 2 | 13.37 (5.36) | 18.94 (10.78) | < 0.001 | 13.21 (5.11) | 15.43 (8.21) | 0.012 |
| Day 3 | 12.68 (5.44) | 16.87 (7.78) | < 0.001 | 12.48 (4.93) | 14.08 (6.84) | 0.075 |
| Day 4 | 13.01 (5.80) | 16.87 (7.73) | 0.0029 | 12.45 (5.48) | 14.64 (6.78) | 0.013 |
| Day 5 | 13.56 (6.08) | 17.54 (6.79) | 0.0020 | 13.20 (6.69) | 15.15 (5.98) | 0.041 |
| Day 6 | 13.39 (5.61) | 19.85 (12.46) | 0.0011 | 13.63 (6.14) | 15.52 (8.97) | 0.45 |
| Day 7 | 19.31 (15.14) | 14.74 (7.02) | 0.058 | 14.58 (6.15) | 16.29 (10.80) | 0.42 |

Table 2 By day of monitoring: mean ICP by outcome at 6 months. ICP denotes intracranial pressure; sd standard deviation

|  | Mean CPP (sd) (mmHg) | | | | |  |
| --- | --- | --- | --- | --- | --- | --- |
|  | Alive | Dead | p | Favourable outcome | Unfavourable outcome | p |
| 1 day | 75.44 (8.02) | 73.90 (12.93) | 0.42 | 75.35 (7.12) | 74.99 (10.60) | 0.47 |
| 3 days | 76.32 (7.07) | 76.30 (13.54) | 0.30 | 76.20 (5.58) | 76.43 (10.46) | 0.34 |
| 5 days | 77.23 (5.74) | 77.98 (12.51) | 0.14 | 77.03 (5.23) | 77.63 (8.72) | 0.44 |
| 7 days | 78.17 (5.18) | 81.68 (8.17) | 0.022 | 77.85 (4.40) | 79.51 (6.83) | 0.082 |

Table 3 By duration of monitoring: mean CPP by outcome at 6 months. CPP denotes cerebral perfusion pressure; sd standard deviation

|  | Mean CPP (sd) (mmHg) | | | | |  |
| --- | --- | --- | --- | --- | --- | --- |
|  | Alive | Dead | p | Favourable outcome | Unfavourable outcome | p |
| Day 1 | 75.44 (8.02) | 73.90 (12.93) | 0.42 | 75.35 (7.12) | 74.99 (10.60) | 0.47 |
| Day 2 | 76.57 (8.70) | 75.36 (14.79) | 0.91 | 76.37 (8.17) | 76.33 (11.58) | 0.21 |
| Day 3 | 77.24 (8.25) | 77.88 (15.31) | 0.25 | 77.23 (7.74) | 77.42 (11.08) | 0.78 |
| Day 4 | 78.86 (9.24) | 78.80 (12.95) | 0.90 | 79.46 (8.09) | 78.33 (11.26) | 0.35 |
| Day 5 | 79.17 (9.32) | 79.70 (9.54) | 0.96 | 79.80 (78.65 (9.40) | 79.80 (9.30) | 0.62 |
| Day 6 | 81.34 (8.10) | 80.46 (17.97) | 0.48 | 80.33 (8.02) | 81.87 (12.63) | 0.090 |
| Day 7 | 81.09 (8.50) | 80.79 (16.90) | 0.21 | 80.01 (7.93) | 81.87 (12.14) | 0.084 |

Table 4 By day of monitoring: mean CPP by outcome at 6 months. CPP denotes cerebral perfusion pressure; sd standard deviation
