# Supplementary material for: Critical thresholds for intracranial pressure vary over time in non-craniectomised traumatic brain injury patients
Source: Acta Neurochir (Wien). 2018 May 7;160(7):1315–24. doi: 10.1007/s00701-018-3555-3 (PMC5996002; doi:10.1007/s00701-018-3555-3)
Supplement: Supplementary file 2 — (DOCX 222 kb) [file 701_2018_3555_MOESM2_ESM.docx]

Appendix B – Sex-Specific Thresholds

In male and female patients, the ICP thresholds for mortality were 21.3 mmHg (χ^2^ = 34.54; p < 0.001) and 24 mmHg (χ^2^ = 15.13; p < 0.001), while the ICP thresholds for unfavourable outcome were 20.7 mmHg (χ^2^ = 14.68; p < 0.001) and 20.1 mmHg (χ^2^ = 5.51; p = 0.0018) respectively.

**Figure 4** Sex-specific thresholds by duration of monitoring. **A.** ICP thresholds in males **B.** ICP thresholds in females. Bold p values remained significant on correction for multiple comparisons. ICP denotes intracranial pressure; NS not significant.

**Figure 5** Sex-specific thresholds by day of monitoring **A.** ICP thresholds in males **B.** ICP thresholds in females. Bold p values remained significant on correction for multiple comparisons. ICP denotes intracranial pressure; NS not significant.
