# Supplementary material for: Critical thresholds for intracranial pressure vary over time in non-craniectomised traumatic brain injury patients
Source: Acta Neurochir (Wien). 2018 May 7;160(7):1315–24. doi: 10.1007/s00701-018-3555-3 (PMC5996002; doi:10.1007/s00701-018-3555-3)
Supplement: Supplementary file 3 — (DOCX 135 kb) [file 701_2018_3555_MOESM3_ESM.docx]

Appendix C

| Duration of monitoring | AUC (95% confidence interval) | | | | |
| --- | --- | --- | --- | --- | --- |
|  | All | Old | Young | Male | Female |
| Whole data | **0.621 (0.565 – 0.677)** | **0.697 (0.57 – 0.825)** | **0.663 (0.59 – 0.736)** | **0.616 (0.556 – 0.676)** | **0.675 (0.525 – 0.824)** |
| 1 day | **0.593 (0.540 – 0.645)** | **0.682 (0.553 – 0.811)** | **0.598 (0.535 – 0.662)** | **0.567 (0.517 – 0.617)** | **0.729 (0.564 – 0.894)** |
| 3 days | **0.653 (0.577 – 0.728)** | 0.658 (0.486 – 0.832) | **0.652 (0.563 – 0.741)** | **0.562 (0.506 – 0.618)** | **0.703 (0.516 – 0.891)** |
| 5 days | **0.669 (0.59 – 0.748)** | 0.593 (0.336 – 0.850) | **0.693 (0.614 – 0.772)** | **0.667 (0.580 – 0.754)** | 0.757 (0.549 – 0.965) |
| 7 days | **0.624 (0.536 – 0.712)** | 0.621 (0.289 – 0.954) | **0.639 (0.542 – 0.736)** | 0.614 (0.515 – 0.713) | 0.679 (0.428 – 0.930) |

Table 1 By duration of monitoring: AUC of univariable logistic regression analysis for ICP threshold for mortality at 6 months. Bold values indicate results were the ICP threshold was significant. AUC denotes area under receiver operating characteristic curve; ICP intracranial pressure

| Duration of monitoring | AUC of logistic regression analysis for ICP threshold for favourable vs unfavourable outcome (95% confidence interval) | | | | |
| --- | --- | --- | --- | --- | --- |
|  | All | Old | Young | Male | Female |
| Whole data | **0.576 (0.546 – 0.606)** | **0.596 (0.539 – 0.653)** | **0.581 (0.5455 – 0.617)** | **0.569 (0.537 – 0.600)** | **0.608 (0.534 – 0.682)** |
| 1 day | **0.574 (0.542 – 0.605)** | **0.652 (0.550 – 0.755)** | **0.582 (0.543 – 0.620)** | **0.572 (0.541 – 0.604)** | **0.568 (0.506 – 0.630)** |
| 3 days | **0.559 (0.524- 0.593)** | 0.604 (0.518 – 0.691) | **0.593 (0.529 – 0.657)** | **0.565 (0.531 – 0.599)** | 0.605 (0.497 – 0.713) |
| 5 days | **0.569 (0.529 – 0.608)** | 0.579 (0.495 – 0.663) | **0.618 (0.547 – 0.690)** | **0.584 (0.543 – 0.625)** | 0.621 (0.487 – 0.754) |
| 7 days | 0.575 (0.498 – 0.652) | 0.679 (0.548 – 0.809) | **0.593 (0.510 – 0.677)** | **0.580 (0.513 – 0.648)** | 0.507 (0.38 – 0.634) |

Table 2 By duration of monitoring: AUC of univariable logistic regression analysis for ICP threshold for favourable vs unfavourable outcome at 6 months. Bold values indicate results were the ICP threshold was significant. AUC denotes area under receiver operating characteristic curve; ICP intracranial pressure

Table 3 By day of monitoring: AUC of univariable logistic regression analysis for ICP threshold for mortality at 6 months. Bold values indicate results were the ICP threshold was significant. AUC denotes area under receiver operating characteristic curve; ICP intracranial pressure

| Day of monitoring | AUC of logistic regression analysis for ICP threshold for alive vs dead (95% confidence interval) | | | | |
| --- | --- | --- | --- | --- | --- |
|  | All | Old | Young | Male | Female |
| Day 1 | **0.593 (0.540 – 0.645)** | **0.682 (0.553 – 0.811)** | **0.598 (0.535 – 0.662)** | **0.567 (0.517 – 0.617)** | **0.729 (0.564 – 0.894)** |
| Day 2 | **0.701 (0.629 – 0.772)** | **0.809 (0.690 – 0.929)** | **0.707 (0.624 – 0.790)** | **0.706 (0.627 – 0.786)** | **0.687 (0.513 – 0.861)** |
| Day 3 | **0.676 (0.592 – 0.760)** | **0.636 (0.598 – 0.774)** | **0.730 (0.647 – 0.812)** | **0.667 (0.576 – 0.758)** | **0.785 (0.616 – 0.954)** |
| Day 4 | **0.650 (0.562 – 0.737)** | 0.562 (0.44 – 0.685) | **0.689 (0.593 – 0.784)** | **0.611 (0.514 – 0.708)** | **0.812 (0.658 – 0.966)** |
| Day 5 | **0.665 (0.572 – 0.758)** | 0.604 (0.366 – 0.842) | **0.699 (0.597 – 0.802)** | **0.672 (0.572 – 0.773)** | **0.736 (0.451 – 1)** |
| Day 6 | **0.694 (0.601 – 0.787)** | 0.758 (0.422 – 1) | **0.586 (0.516 – 0.656)** | **0.702 (0.602 – 0.803)** | 0.742 (0.484 – 1) |
| Day 7 | **0.621 (0.511 – 0.731)** | 0.667 (0.321 – 1) | **0.642 (0.526 – 0.759)** | 0.613 (0.493 – 0.734) | 0.741 (0.645 – 0.837) |

| Day of monitoring | AUC of logistic regression analysis for ICP threshold for favourable vs unfavourable outcome (95% confidence interval) | | | | |
| --- | --- | --- | --- | --- | --- |
|  | All | Old | Young | Male | Female |
| Day 1 | **0.574 (0.542 – 0.605)** | **0.652 (0.550 – 0.755)** | **0.582 (0.543 – 0.620)** | **0.572 (0.541 – 0.604)** | **0.568 (0.506 – 0.630)** |
| Day 2 | **0.585 (0.541 – 0.631)** | **0.671 (0.562 – 0.780)** | **0.598 (0.538 – 0.657)** | **0.610 (0.558 – 0.661)** | 0.573 (0.459 – 0.689) |
| Day 3 | **0.583 (0.540 – 0.626)** | **0.618 (0.545 – 0.69)** | **0.582 (0.531 – 0.633)** | **0.569 (0.527 – 0.611)** | 0.613 (0.509 – 0.717) |
| Day 4 | **0.596 (0.534 – 0.658)** | 0.629 (0.517 – 0.740) | **0.608 (0.540 – 0.677)** | **0.597 (0.524 – 0.670)** | 0.647 (0.524 – 0.771) |
| Day 5 | **0.570 (0.518 – 0.622)** | 0.597 (0.423 – 0.771) | **0.582 (0.524 – 0.641)** | 0.551 (0.493 – 0.610) | **0.679 (0.537 – 0.82)** |
| Day 6 | 0.528 (0.504 – 0.552) | 0.6 (0.496 – 0.705) | 0.534 (0.505 – 0.563) | 0.534 (0.505 – 0.563) | 0.625 (0.515 – 0.735) |
| Day 7 | 0.565 (0.481 – 0.650) | 0.643 (0.449 – 0.837) | 0.593 (0.503 – 0.684) | 0.554 (0.457 – 0.652) | 0.675 (0.506 – 0.844) |

Table 4 By day of monitoring: AUC of univariable logistic regression analysis for ICP threshold for favourable vs unfavourable outcome at 6 months. Bold values indicate results were the ICP threshold was significant. AUC denotes area under receiver operating characteristic curve; ICP intracranial pressure
