# Supplementary material for: Critical thresholds for intracranial pressure vary over time in non-craniectomised traumatic brain injury patients
Source: Acta Neurochir (Wien). 2018 May 7;160(7):1315–24. doi: 10.1007/s00701-018-3555-3 (PMC5996002; doi:10.1007/s00701-018-3555-3)
Supplement: Supplementary file 4 — (DOCX 210 kb) [file 701_2018_3555_MOESM4_ESM.docx]

Appendix D – Age-Specific Thresholds

**Figure 6** Age-specific thresholds by duration of monitoring **A.** ICP thresholds in young **B.** ICP thresholds in old. Bold p values remained significant on correction for multiple comparisons. ICP denotes intracranial pressure; NS not significant.

**Figure 7** Age-specific thresholds by day of monitoring **A.** ICP thresholds in young patients **B.** ICP thresholds in old patients. Bold p values remained significant on correction for multiple comparisons. ICP denotes intracranial pressure; NS not significant.
