# Supplementary material for: Critical thresholds for intracranial pressure vary over time in non-craniectomised traumatic brain injury patients
Source: Acta Neurochir (Wien). 2018 May 7;160(7):1315–24. doi: 10.1007/s00701-018-3555-3 (PMC5996002; doi:10.1007/s00701-018-3555-3)
Supplement: Supplementary file 5 — (DOCX 120 kb) [file 701_2018_3555_MOESM5_ESM.docx]

APPENDIX E – THRESHOLD TABLES

| Duration of monitoring | ICP threshold for outcome (mmHg) (Chi-square test statistic; p value, sensitivity, specificity) | | | | |
| --- | --- | --- | --- | --- | --- |
|  | All | Old | Young | Male | Female |
| Whole data | 20.5 (20.73; p < 0.001, 17%, 98%) | 19.9 (4.97; p = 0.026, 19%, 100%) | 20.6 (17.35; p < 0.001, 17%, 98%) | 20.7 (14.68; p < 0.001, 15%, 98%) | 20.1 (5.51; p = 0.018, 27%, 95%) |
| 1 day | 22.3 (17.83; p < 0.001, 18%, 97%) | 15.8 (5.82; p = 0.016, 48%, 83%) | 22.3 (15.49; p < 0.001, 20%, 96%) | 22.5 (16.20; p < 0.001, 15%, 99%) | 15 (8.60; p = 0.0034, 65%, 70%) |
| 3 days | 21.2 (9.39; p = 0.0022, 15%, 96%) | 18.7 (3.0; p = 0.083, 75%, 4%) | 13.7 (7.15; p = 0.0075, 69%, 50%) | 21.2 (10.90; p < 0.001, 14%, 99%) | 17.4 (2.43; p = 0.12, 38%, 82%) |
| 5 days | 20.9 (8.54; p = 0.0034, 18%, 96%) | 20.2 (1.01; p = 0.32, 84%, 0%) | 14.7 (9.25; p = 0.0023, 65%, 59%) | 20.9 (10.60; p = 0.0011, 18%, 99%) | 15.9 (2.06; p = 0.15, 46%, 78%) |
| 7 days | 14.8 (3.07; p = 0.080, 61%, 54%) | 10.7 (2.93; p = 0.086, 35%, 100%) | 14.8 (4.02; p = 0.045, 64%, 55%) | 19.5 (3.92; p = 0.048, 27%, 89%) | 16 (0.65; p = 0.42, 58%, 71%) |

By duration of monitoring

| Duration of monitoring | ICP threshold for death (mmHg) (Chi-square test statistic; p value, sensitivity, specificity) | | | | |
| --- | --- | --- | --- | --- | --- |
|  | All | Old | Young | Male | Female |
| Whole data | 21.3 (42.90; p < 0.001, 30%, 97%) | 14.7 (8.3; p = 0.0039, 61%, 78%) | 20.7 (39.34; p < 0.001, 32%, 95%) | 21.3 (34.54; p < 0.001, 25%, 98%) | 24 (15.13; p < 0.001, 36%, 99%) |
| 1 day | 24.7 (31.30; p < 0.001, 20%, 98%) | 17.9 (8.89; p = 0.0029, 47%, 89%) | 24.7 (25.15; p < 0.001, 21%, 98%) | 24.7 (17.17; p < 0.001, 14%, 99%) | 25.1 (15.56, p < 0.001, 50%, 96%) |
| 3 days | 15.2 (13.16; p < 0.001, 68%, 61%) | 14.6 (2.4; p = 0.12, 60%, 72%) | 17.7 (11.8; p < 0.001, 54%, 76%) | 24.6 (11.02; p < 0.001, 14%, 99%) | 17.9 (3.98; p = 0.046, 63%, 78%) |
| 5 days | 15.1 (12.70; p < 0.001, 76%, 58%) | 13.1 (0.082; p = 0.77, 60%, 59%) | 15.1 (14.2; p < 0.001, 81%, 57%) | 15.1 (10.29; p = 0.0013, 75%, 58%) | 15.9 (3.22; p = 0.072, 80%, 71%) |
| 7 days | 14.1(4.89; p = 0.026, 79%, 46%) | 10 (0.070; p = 0.79, 66%, 9%) | 15 (5.27; p = 0.021, 76%, 52%) | 14.1 (3.16; p = 0.075, 78%, 45%) | 19.3 (1.24; p = 0.27, 60%, 76%) |

By day

| Day of monitoring | ICP threshold for death (mmHg) (Chi-square test statistic; p value, sensitivity, specificity) | | | | |
| --- | --- | --- | --- | --- | --- |
|  | All | Old | Young | Male | Female |
| Day 1 | 24.7 (31.30; p < 0.001, 20%, 98%) | 17.9 (8.89; p = 0.0029, 47%, 89%) | 24.7 (25.15; p < 0.001, 21%, 98%) | 24.7 (17.17; p < 0.001, 14%, 99%) | 25.1 (15.56, p < 0.001, 50%, 96%) |
| Day 2 | 16.5 (29.49; p < 0.001, 65%, 75%) | 15.1 (16.41; p < 0.001, 80%, 82%) | 16.5 (21.26; p < 0.001, 69%, 72%) | 16.5 (25.29; p < 0.001, 64%, 77%) | 15.5 (5.99; p = 0.014, 78%, 71%) |
| Day 3 | 16.6 (18.40; p < 0.001, 58%, 77%) | 22.3 (8.15; p = 0.0043, 27%, 100%) | 15.0 (18.54; p < 0.001, 81%, 64%) | 14.3 (10.45; p = 0.0012, 70%, 63$) | 17.7 (8.90; p = 0.0028, 75%, 82%) |
| Day 4 | 15.7 (10.47; p = 0.0012, 61%, 69%) | 28.3 (0.61; p = 0.44, 88%, 0%) | 15.7 (13.10; p < 0.001, 68%, 70%) | 18.4 (5.56; p = 0.018, 41%, 81%) | 15.9 (8.11; p = 0.0044, 86%, 77%) |
| Day 5 | 19.2 (14.60; p < 0.001, 50%, 83%) | 15 (0.23; p = 0.63, 50%, 71%) | 19.2 (16.49; p < 0.001, 58%, 82%) | 16.8 (10.66; p = 0.0011, 61%, 74%) | 24.3 (5.58; p = 0.018, 50%, 97%) |
| Day 6 | 18.6 (18.69; p < 0.001, 56%, 83%) | 18.7 (1.62; p = 0.20, 66%, 85%) | 29.7 (15.30; p < 0.001, 17%, 100%) | 18.6 (16.93; p < 0.001, 57%, 83%) | 16.9 (1.88; p = 0.17, 75%, 73%) |
| Day 7 | 18.7 (4.37; p = 0.037, 50%, 74%) | 18.7 (0.21; p = 0.65, 67%, 67%) | 19.7 (6.09; p = 0.014, 48%, 81%) | 19.7 (3.17; p = 0.075, 45%, 78%) | 12.1 (1.63; p = 0.20, 100%, 48%) |

| Day of monitoring | ICP threshold for outcome (mmHg) (Chi-square test statistic; p value, sensitivity, specificity) | | | | |
| --- | --- | --- | --- | --- | --- |
|  | All | Old | Young | Male | Female |
| Day 1 | 22.3 (17.83; p < 0.001, 18%, 97%) | 15.8 (5.82; p = 0.016, 48%, 83%) | 22.3 (15.49; p < 0.001, 20%, 96%) | 22.5 (16.20; p < 0.001, 15%, 99%) | 15 (8.60; p = 0.0034, 65%, 70%) |
| Day 2 | 18.8 (12.41; p < 0.001, 28%, 89%) | 15.1(5.87; p = 0.015, 47%, 87%) | 16.7 (9.16; p = 0.0025, 43%, 77%) | 18.4 (14.97; p < 0.001, 32%, 90%) | 15.5 (1.00; p = 0.32, 43%, 72%) |
| Day 3 | 19.1 (16.30; p < 0.001, 24%, 92%) | 17.4 (4.27; p = 0.039, 24%, 100%) | 19.1 (8.67; p = 0.0032, 26%, 91%) | 20.0 (8.75; p = 0.0031, 18%, 96%) | 19.1 (3.06; p = 0.080, 33%, 89%) |
| Day 4 | 15.2 (7.96; p = 0.0048, 47%, 72%) | 19 (2.42; p = 0.12, 32%, 94%) | 15.2 (8.21; p = 0.0041, 49%, 73%) | 12.9 (5.83; p = 0.016, 63%, 56%) | 15.9 (3.72; p = 0.053, 46%, 83%) |
| Day 5 | 20 (5.44; p = 0.020, 23%, 91%) | 14.9 (0.48; p = 0.49, 44%, 75%) | 20 (6.02; p = 0.014, 26%, 91%) | 20.2 (2.05; p = 0.15, 21%, 89%) | 16.8 (5.68; p = 0.017, 56%, 81%) |
| Day 6 | 29.7 (2.65; p = 0.10, 5%, 100%) | 20.3 (0.50; p = 0.48, 80%, 0%) | 29.7 (2.92; p = 0.087, 7%, 100%) | 29.7 (2.42; p = 0.12, 93%, 0%) | 20.6 (2.98; p = 0.085, 25%, 100%) |
| Day 7 | 14.8 (1.79; p = 0.18, 57%, 56%) | 10.3 (0.67; p = 0.41, 43%, 86%) | 15.6 (3.27; p = 0.071, 57%, 62%) | 15.6 (0.82; p = 0.36, 54%, 57%) | 12.1 (2.59; p = 0.11, 75%, 60^) |
